# Antimicrobial activity of sesquiterpene lactones isolated from traditional medicinal plant, *Costus speciosus *(Koen ex.Retz.) Sm

**DOI:** 10.1186/1472-6882-12-13

**Published:** 2012-03-07

**Authors:** Veeramuthu Duraipandiyan, Naif Abdullah Al-Harbi, Savarimuthu Ignacimuthu, Chinnasamy Muthukumar

**Affiliations:** 1 Department of Botany and Microbiology, Addiriyah Chair for Environmental Studies, College of Science, King Saud University, P. O. Box 2455, Riyadh 11451, Saudi Arabia; 2 Division of Ethnopharmacology, Entomology Research Institute, Loyola College, Chennai 600 034, India

**Keywords:** Antibacterial, Antifungal, Sesquiterpeneoids, *Costus speciosus*, Costunolide, Eremanthin, MIC

## Abstract

**Background:**

*Costus speciosus *(Koen ex.Retz.) Sm (Costaceae) is an Indian ornamental plant which has long been used medicinally in traditional systems of medicine. The plant has been found to possess diverse pharmacological activities. Rhizomes are used to treat pneumonia, rheumatism, dropsy, urinary diseases, jaundice, skin diseases and leaves are usedto treat mental disorders.

**Method:**

Antibacterial and antifungal activities were tested using Disc diffusion method and Minimum Inhibitory **Concentration **(MIC). Column chromatography was used to isolate compounds from hexane extract. X-ray crystallography technique and GC-MS analysis were used to identify the compounds

**Results:**

Antibacterial and antifungal activities were observed in hexane, chloroform, ethyl acetate and methanol extracts. Hexane extract of *C.speciosus *showed good activity against tested fungi also. Two sesquiterpenoid compounds were isolated (costunolide and eremanthin) from the hexane extract. Both the compounds did not inhibit the growth of tested bacteria. But, both the compounds inhibited the tested fungi. The compound costunolide showed significant antifungal activity. The MIC values of costunolide were; 62.5 μg/ml against *Trichophyton mentagrophytes*, 62. μg/ml against *T. simii*, 31.25 μg/ml against *T. rubrum *296, 62.5 μg/ml against *T. rubrum *57, 125 μg/ml against *Epidermophyton floccosum*, 250 μg/ml against *Scopulariopsis *sp, 250 μg/ml against *Aspergillus niger*, 125 μg/ml against *Curvulari lunata*, 250 μg/ml against *Magnaporthe grisea*.

**Conclusion:**

Hexane extract showed promising antibacterial and antifungal activity. The isolated compound costunolide showed good antifungal activity.

## Background

Infectious diseases caused by bacteria, fungi, viruses and parasites are still a major threat to public health, despite the tremendous progress in human medicine. Their impact is particularly large in developing countries due to the relative unavailability of medicines and the emergence of widespread drug resistance [1]. Medicinal plants have been a source of bioactive compounds to treat many diseases. Traditionally used medicinal plants produce a variety of compounds with known therapeutic properties [2].

*Costus speciosus *(Koen ex.Retz.) Sm. (Costaceae, Family)is widely used in Ayurveda. The plant rhizome is bitter, useful in treating burning sensation, constipation, leprosy, worm infection, skin diseases, fever, asthma, bronchitis, inflammations and anaemia [3,4].

*C. speciosus *leaf infusion or decoction is utilized as a sudorific or in a bath for patients with high fever. Rhizome juice is given with sugar internally to treat leprosy [5]. *C. speciosus *rhizomes and roots are ascribed to be bitter [6], astringent [7,8], acrid, cooling, aphrodisiac [9,10], purgative and depurative [5], anthelmintic [8,9], antituberculosis [11], spermatorrhoea [12] and antioxidant [13]. Juice of the rhizome is applied on the head for cooling and relief from headache [7]. An alkaloid extracted from *C. speciosus *rhizomes had papaverine-like smooth muscle relaxant and antispasmodic activities [14]. *C. spec*iosus rhizomes are given to treat pneumonia, rheumatism, dropsy, urinary diseases, jaundice and leaves are given to treat mental disorders; bruised leaves are applied topically to reduce fever; decoction of stem is used to control fever and dysentery [15].

Gupta et al. [16] reported five compounds from the rhizomes of *C. speciosus *tetradecyl **13-**methylpentadecanoate, tetradecyl -11-methyltridecanoatc, 14-oxotricosanoic acid, 14-oxoheptacosanoic acid and 15-oxo-octacosanoic acid. Triacontanol, 5α-stigmast-9 (11) en -3β-ol, triacontanoic acid, sitosterol and diosgenin have also been isolated and identified.

Costunolide is a sesquiterpene compound. It has been previously isolated from *Saussurea radix *and the dried root of *S. lappa*. It is reported to possess various biological and immunological actions [17-19]. Some kinds of sequiterpene compounds induced apoptosis in cancer cells [20,21], and costunolide also exhibited preventive effects on intestinal carcinogenesis [22]. Sesquiterpene such as costunolide isolated from the leaves of *Laurus nobilis *was found to potently inhibit blood ethanol elevation in ethanol loaded rats [23,24]. Previously eremanthin was reported to be present in *Pterodon pubescens*, *Eremanthus elaeagnus *[25], and n-hexane extract of *Larus nobilis *leaves [26].

We have already reported the hypoglycemic effect of *C. speciosus *root crude extracts [27], hypolipidemic effect of costunolide [3], antidiabetic and antilipidemic effect of eremanthin [28] and antioxidant activities of costunolide and eremanthin [29]. We herein report the antimicrobial activity of the crude extracts and two compounds from the rhizome of *C*. *speciosus*****againstpathogenic bacteria and fungi.

## Methods

### Plant material

The rhizomes of *Costus speciosus *(Koen ex.Retz.) were collected from Thandarai, Kanchipuram District, Tamil Nadu, India in 2005. The plant was identified by Dr. D. Narasimman, Department of Botany, Madras Christian College, Chennai, India. The voucher specimen (ERIC-D-78) was deposited at Entomology Research Institute, Loyola College, Chennai, India.

### Preparation of extracts

The collected rhizomes were shade dried at room temperature and ground in a manual mill. Two kilogram powder was extracted with 6 litre of hexane (1:3; w/v) for 48 hours. The extract was filtered through a Buchner funnel with Whatman number 1 filter paper. The filtrate was evaporated to dryness under reduced pressure using rotary evaporator at 40°C. The remains of the plant material were extracted using chloroform, ethyl acetate, methanol and water sequentially in a similar manner using cold percolation method [30]. The yields of extracts were: hexane (25 g); chloroform (22 g); ethyl acetate (28 g), methanol (21 g) and water (19 g).

### Isolation and identification of the active compound

One kg of coarse powder was soaked in 3 litres of hexane for 72 hrs with intermittent shaking. The filtrate was filtered through Buchner funnel and concentrated using vacuum rotary evaporator at 40°C. 25 g of active crude hexane extract was chromatographed on a silica gel column (Merk 10-200 mesh, 750 gm 3.5 i.d × 60 cm) and successively eluted with stepwise gradient of petroleum ether and hexane solvent system (5%, 10%, 20%, 30%, 50%, 70% and 100%). 116 fractions were collected and each fraction was spotted on a precoated silica gel (Merk-60 F254, 0.25 mm thick) plate and eluted in Hexane: Ethyl acetate(3:1) and fractions with similar Rf values in TLC pattern were pooled together to get 8 fractions. Fraction 3 was rechromatographed on a silica gel column and eluted with a stepwise gradient of Hexane: Ethyl acetate (9:1) solvent system. An oil substance was obtained in subfraction 1; it was identified as eremanthin with a molecular formula C_15_H_18_O_2_, molecular weight of m/z 230.13, m.p 72-75°C and Rf value 0.35. Based on the GC-MS analysis the fraction 1 was confirmed as Eremanthin [28].

A single pure crystal was obtained in sub fraction 3 eluted with a stepwise gradient of Hexane: Ethyl acetate (8:2) solvent system. The crystal was subjected to crystallographic and spectral analysis for structural determination. The X-ray data for crystal were recorded using Bruker-AX, X-ray diffractometer at the Indian Institute of Technology, Chennai, India. The compound was identified as costunolide [3], with molecular formula C_15_H_20_O_2_, molecular weight m/z 234.33 and m.p 105-108°C. The Rf value of costunolide was 0.40.

### Tested microorganisms

The following bacteria and fungi were used for the experiment. Bacteria: *Bacillus subtilis *MTCC 441, *Enterococcus faecalis *ATCC 29212, *Staphylococcus aureus *ATCC 25923, *S. epidermidis *MTCC 3615, *Escherichia coli *ATCC 25922, *Klebsiella pneumoniae *ATCC 15380, *Proteus vulgaris *MTCC 1771, *Pseudomonas aeruginosa *ATCC 27853 and *Erwinia *sp. MTCC 2760; Fungi: *T. rubrum *MTCC 296, *T. rubrum *57/01, *T. mentagrophytes *66/01, *T. simii *110/02, *Epidermophyton floccosum *73/01, *Scopulariopsis *sp. 101/01 *Aspergillus niger *MTCC 1344, *Botyritis cinerea, Curvularia lunata *46/0, *Magnoporthe grisea *and *Candida albicans *MTCC 227. All cultures were obtained from the Department of Microbiology, Christian Medical College, Vellore, Tamil Nadu, India.

### Preparation of inoculums

The mother culture was streaked on Nutrient Agar medium to obtain isolated colonies. After incubation at 37°C 24 h, 4 or 5 pure colonies were selected with an inoculating needle and transferred to a tube of sterile Mueller-Hinton broth and vortexed thoroughly. The bacterial suspension was equal to the 0.5 McFarland standards. These cell suspensions were diluted with sterile MHB to provide final cell counts of about 10^4 ^CFU/ml. The filamentous fungi were grown on Sabouraud Dextrose Agar (SDA) slants at 28°C for 10 days and the spores were collected using sterile doubled distilled water and homogenized. Yeast was grown on Sabouraud Dextrose Broth (SDB) at 28°C for 48 h.

### Disc diffusion assay

Antibacterial activity was carried out for crude extracts using disc-diffusion method [30]. Petri plates were prepared with 20 ml of sterile Mueller Hinton Agar (MHA) (Hi-media, Mumbai). The test cultures were swabbed on the top of the solidified media and allowed to dry for 10 min. The tests were conducted at three different concentrations of the crude extract (5 mg, 2.5 mg and 1.25 mg per disc). The loaded discs were placed on the surface of the medium and left for 30 min at room temperature for compound diffusion. Negative control was prepared using respective solvent. Streptomycin (10 μg/disc) was used as positive control. The plates were incubated for 24 h at 37°C. Zones of inhibition were recorded in millimeters and the experiment was repeated twice.

### Minimum Inhibitory Concentration (MIC)

The antifungal activity and Minimum Inhibitory Concentration (MIC) were performed using microdilution technique according to the standard reference method [30]. The extracts were dissolved in water + 2% Dimethyl Sulfoxide (DMSO). The initial concentration of the extract was 1 mg/ml. Test concentration was serially diluted two-fold in a 96 well plate. Each well was inoculated with 5 μl of suspension containing 10^4 ^CFU/ml of bacteria and 10^4 ^spore/ml of fungi, respectively. For fungi, the plates were incubated for 24, 48 or 72 h at 27° up to 9 days for dermatophytes strains; bacteria plates were incubated for 24 h at 37°C. MIC was defined as the lowest extract concentration, showing no visible fungal growth after incubation time. 5 μl of tested broth was placed on the sterile MHA plates for bacteria and incubated at respective temperature. The MIC for bacteria was determined as the lowest concentration of the compound inhibiting the visual growth of the test cultures on the agar plate.

### Antifungal activity of compounds

The antifungal activity of costunolide and eremanthin isolated from the hexane extract of the rhizome of *C. speciosus *was assessed using standard reference method [30]. 250 μg/ml of the compound was dissolved in water + 2% Dimethyl Sulfoxide (DMSO). It was serially diluted two-fold. Each well was inoculated with 5 μl of suspension containing 10^4 ^spore/ml of fungi. The antifungal agents' Ketoconazole and Fluconazole were included in the assays as positive controls.

## Results

Hexane, chloroform, ethyl acetate, methanol and water extracts were screened against bacteria and fungi using disc diffusion method and microdilution technique. Hexane extract inhibited the growth of *S. aureus *(15 mm) at 2.5 mg/disc, *S. epidermidis *(15 mm) at 5 mg/disc and *B. subtilis *(12 mm) at 2.5 mg/disc. Chloroform extract inhibited the growth of *S. aureus *(12 mm) at 2.5 mg/disc, *S. epidermidis *(13 mm) at 2.5 mg/disc and *B. subtilis *(9 mm) at 5 mg/disc. Ethyl acetate extract inhibited the growth of *S. aureus *(10 mm) at 5 mg/disc, *S. epidermidis *(9 mm) at 5 mg/disc and *B. subtilis *(9 mm) at 5 mg/disc. Methanol extract showed activity against *S. aureus *(14 mm) at 2.5 mg/disc, *S. epidermidis *(12 mm) at 2.5 mg/disc and *B. subtilis *(10 mm) at 5 mg/disc. Among the tested extracts the hexane extract showed good activity against only Gram-positive bacteria and tested fungi (Table 1).

**Table 1:** Minimum inhibitory concentration of different crude extracts of *Costus speciosus *rhizome against selected bacteria and fungi

Tested organisms	Minimum inhibitory concentration (mg/ml)				
**Bacteria**	**He**	**Ch**	**E.a**	**Me**	**W**

*Staphylococcus aureus*	1.25	0.625	1.25	0.625	> 5
*Staphylococcus epidermidis*	0.625	0.312	5	0.625	> 5
*Bacillus subtilis*	1.25	> 5	> 5	1.25	> 5

**Fungi**	**Minimum inhibitory concentration (μg/ml)**				

*Trichophyton mentagrophytes*	0.62	1000	0.62	1000	1000
*Epidermophyton floccosum *	0.62	1000	0.62	0.125	1000
*Trichophyton simii*	0.62	> 1000	0.62	1000	> 1000
*Trichophyton rubrum *296	0.62	1000	0.125	500	1000
*Trichophyton rubrum *57/01	0.125	500	0.125	0.125	1000
*Curvularia lunata*	500	1000	0.250	> 1000	> 1000
*Aspergillus niger*	500	> 1000	> 1000	> 1000	> 1000
*Botrytis cinerea*	1000	> 1000	> 1000	> 1000	> 1000
*Scopulariopsis *sp.	500	> 1000	> 1000	500	> 1000
*Magnaporthe grisea*	0.125	> 1000	1000	> 1000	> 1000
*Candida albicans*	> 1000	> 1000	> 1000	> 1000	> 1000

He-Hexane extract; Ch-Chloroform extract; E.a-Ethyl acetate extract; Me -Methanol extract; W-Water extract

Among the tested extracts for antifungal activity the hexane extract significantly inhibited the growth of *T. mentagrophytes *at 0.125 mg/ml, *E. floccosum *at 0.250 mg/ml, *T. rubrum *at 0.125 mg/ml and *M. grisea *at 0.125 mg/ml. Other extracts inhibited the growth of fungi moderately. The results are summarized in Table 1.

**Figure 1:**
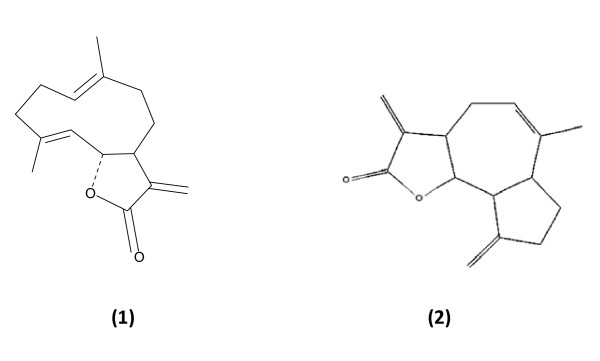
**Costunolide (1) and Eremanthin (2)**.

Based on the above results the hexane extract was subjected to fractionation. Two active sesquiterpenoids, costunolide and eremanthin, were isolated from hexane extract of *C. speciosus *(Figure 1).

The compounds costunolide and eremanthin were tested against bacteria and fungi using micro broth dilution method. Both the compounds inhibited the tested fungi but not the bacteria. The compound costunolide inhibited the growth of fungi such as *T. mentagrophytes *(62.5 μg/ml), *T. simii *(31.25 μg/ml), *T. rubrum *296 (31.25 μg/ml), *T. rubrum *57 (62.5 μg/ml), *E. floccosum *(125 μg/ml), *Scopulariopsis *sp (250 μg/ml), *A. niger *(250 μg/ml), *C. lunata *(125 μg/ml) and *M. grisea *(250 μg/ml) (Table 3). The compound eremanthin inhibited the growth of *T. mentagrophytes *(125 μg/ml), *T. simii *(62.5 μg/ml), *T. rubrum *296 (62.5 μg/ml), *T. rubrum *57 (250 μg/ml), *E. floccosum *(125 μg/ml), *A. niger *(125 μg/ml), *C. lunata *(250 μg/ml) and *M. grisea *(250 μg/ml). Both the compounds showed almost similar **activities **against fungi (Table 2). The lowest MIC values were obtained in costunolide against *T. simii *at 62.5 μg/ml and *T. rubrum *296 at 62.5 μg/ml.

**Table 2:** Minimum Inhibitory concentrations of costunolide and eremanthin isolated from *Costus speciosus *against tested fungi using microdilution technique (μg/ml)

Tested organisms	Costunolide	Eremanthin	Fluconazole	Ketoconazole
*Trichophyton mentagrophytes*	62.5	125	25	< 12.5
*Trichophyton simii*	31.25	62.5	< 12.5	< 12.5
*Trichophyton rubrum *296	31.25	62.5	< 12.5	< 12.5
*Trichophyton rubrum *57	62.5	250	25	< 12.5
*Epidermophyton floccosum*	125	125	12.5	< 12.5
*Scopulariopsis *sp.	250	> 250	< 12.5	< 12.5
*Aspergillus niger*	250	125	100	< 12.5
*Curvularia lunata*	125	250	< 12.5	< 12.5
*Magnaporthe grisea*	250	250	nt	nt
*Botrytis cinerea*	> 250	> 250	nt	nt
*Candida albicans*	> 250	> 250	> 100	25

nt-not Tested

Fluconazole, Ketoconazole-standard antifungal agent

Costunolide, Eremanthin-Isolated compound

## Discussion

Sesquiterpene lactones are the most distinctive secondary metabolites of the members of the Compositae (Asteraceae). However, they have been reported from several plant families, such as Acanthaceae, Amaranthaceae, Apiaceae, Magnoliaceae, Costaceae. They have a diversity of chemical structures and a wide range of biological activities, including antitumourogenic, insect antifeedant, plant growth regulating, antibacterial, antifungal and cytotoxic properties [31-35]. Here we report the antibacterial and antifungal activities of the extracts of *C. speciosus *rhizome. The hexane extract of *C. speciosus *showed significant antifungal activity. Saraf [36] reported that methanol and aqueous extracts of *C. specious *rhizome did not exhibit any antimicrobial activity against *E. coli, S. aureus*, *K. pnuemoniae, P. aeruginosa*. In this study, water extract did not inhibit any tested bacteria; however methanol extract inhibited the growth of Gram-positive bacteria such as *S. aureus. S. epidermidis *and *B. subtilis*. Swarnkar and Katewa [37] reported that methanol and water extracts of *C. speciosus *rhizome inhibited the growth of *S. aureus *(12 mm). Our study showed that methanol extract inhibited the growth of *S. aureus *(12 mm), *S. epidermidis *(9 mm) and *B. subtilis *(14 mm). Chen et al. [18] reported that methanol extract of *C. speciosus *showed activity against *E. coli*, *Salmonella enterica *and *S. aureus*.

The isolated compounds, costunolide and eremanthin, significantly inhibited the tested pathogenic fungi at lowest concentrations. Ahmed and Abdelgalei [38] reported that costunolide isolated from *Magnolia grandiflora *bark showed antifungal activity against plant pathogenic fungi. They found that costunolide showed higher antifungal activity than the reference control.

The present study also showed costunolide inhibited the growth of tested fungi better than eremanthin. Both the compounds were isolated using low polar solvent (hexane). Barrero *et al*. [39] stated that low polar sesquiterpene lactones showed more potent antifungal activity. It is well known that the presence of α-methylene-γ-lactone is essential for potent antifungal activity of sesquiterpene lactones. The low polarity of these compounds matches with optimum lipophilic characteristics required for passing through the fungal cell wall [38].

Antifungal activity of steroid saponins and sapogenins from *C. speciosus *was analyzed previously by Singh *et al*. [40] on six species of plant pathogenic fungi at different concentrations. Saponin B was found to be highly effective against conidial germination of *Botrytis cineria *and *Alternaria *sp.

p-Coumaric acid methyl ester was found in the rhizome of *C. speciosus *as a constitutive principle with antifungal activity against plant pathogenic fungi *Cladosporium cladosporioides, Colletotrichum gleosporioides, Curvularia *sp. and *Penicillium *sp. [41]. Moreira *et al*. [42] have reported that sesquiterpenoids (aromadendrane-4β, 10α,15-triol) isolated from the leaves of *Xylopia brasiliensis *showed activity against *Cladosporium cladosporioides*. In our study costunolide and eremanthin showed good antifungal activity.

## Conclusion

A thorough analysis of the results indicated that among the extracts of *C. speciosus*, only the hexane extract showed moderate activity against tested bacteria and promising activity against fungi. Two compounds, costunolide and eremanthin, were isolated from the hexane extract of *C. speciosus *with significant antifungal activity *in vitro*. This is the first report for the antifungal activity of costunolide and eremanthin against dermatophytes.

## Competing interests

The authors declare that they have no competing interests.

## Authors' contributions

VD and CM carried out the study; VD designed the experiments and wrote the manuscript; SI and NAAH supervised the work; all authors read and approved the final manuscript.

## Pre-publication history

The pre-publication history for this paper can be accessed here:

http://www.biomedcentral.com/1472-6882/12/13/prepub
